# Optimized Generalized LDPC Convolutional Codes

**DOI:** 10.3390/e27090930

**Published:** 2025-09-04

**Authors:** Li Deng, Kai Tao, Zhiping Shi, You Zhang, Yinlong Shi, Jian Wang, Tian Liu, Yongben Wang

**Affiliations:** 1 Yangtze Delta Region Institute (Huzhou), University of Electronic Science and Technology of China, Huzhou 313000, China; dengli@uestc.edu.cn; 2 National Key Laboratory on Communications, University of Electronic Science and Technology of China, Chengdu 611731, China; 202411220621@std.uestc.edu.cn; 3 ZTE Corporation, Shenzhen 518057, China; tao.kai@zte.com.cn (K.T.); shi.yinlong@zte.com.cn (Y.S.); wang.jian59@zte.com.cn (J.W.); liu.tian2@zte.com.cn (T.L.); wang.yongben@zte.com.cn (Y.W.)

**Keywords:** generalized LDPC convolutional code, doping, Chase–Pyndiah, hybrid layered normalized min-sum, adaptive weighting factor, low decoding complexity

## Abstract

In this paper, some optimized encoding and decoding schemes are proposed for the generalized LDPC convolutional codes (GLDPC–CCs). In terms of the encoding scheme, a flexible doping method is proposed, which replaces multiple single parity check (SPC) nodes with one generalized check (GC) node. Different types of BCH codes can be selected as the GC node by adjusting the number of SPC nodes to be replaced. Moreover, by fine-tuning the truncated bits and the extended parity check bits, or by reasonably adjusting the GC node distribution, the performance of GLDPC–CCs can be further improved. In terms of the decoding scheme, a hybrid layered normalized min-sum (HLNMS) decoding algorithm is proposed, where the layered normalized min-sum (LNMS) decoding is used for SPC nodes, and the Chase–Pyndiah decoding is adopted for GC nodes. Based on analysis of the decoding convergence of GC node and SPC node, an adaptive weight factor is designed for GC nodes that changes as the decoding iterations, aiming to further improve the decoding performance. In addition, an early stop decoding strategy is also proposed based on the minimum amplitude threshold of mutual information in order to reduce the decoding complexity. The simulation results have verified the superiority of the proposed scheme for GLDPC–CCs over the prior art, which has great application potential in optical communication systems.

## 1. Introduction

The evolution of forward error correction (FEC) in optical communication systems has generally progressed through three generations, with different standards recommending distinct FEC codes over time. The first-generation FEC employed hard-decision codes, notably the RS (255,239) code specified in standards like ITU-T G.975 and G.709, which provided approximately 6 dB of net coding gain (NCG) and was primarily used in synchronous digital hierarchy (SDH) and early wavelength division multiplexing (WDM) systems. As single-wavelength 10G/40G WDM systems demanded higher error correction capabilities, second-generation FEC utilized hard-decision concatenated coding techniques (such as the concatenated codes defined in ITU-T G.975.1), achieving NCG above 8 dB through interleaving and iterative decoding. With the advent of single-wavelength 100G and beyond-100G coherent systems, the development of coherent technology and high-speed integrated circuits facilitated the adoption of third-generation soft-decision FEC (SD-FEC), where LDPC codes (low-density parity check codes) and turbo product codes (TPC) became mainstream, offering approximately 11 dB of NCG and significantly enhancing long-haul transmission performance. Owing to their near-Shannon-limit performance, low decoding complexity, and high throughput potential, LDPC codes have been widely incorporated into optical communication standards, such as 100G/400G/800G coherent transmission systems (e.g., OIF 400ZR/800ZR and ITU-T G.709.3 FlexO interfaces), which utilize LDPC-based codes.

Generalized low-density parity check (GLDPC) codes are a class of channel codes with broad code rate selection and performance approaching the Shannon limit. Compared to conventional low-density parity check (LDPC) codes, GLDPC codes replace single parity check (SPC) nodes with generalized constraint (GC) nodes of stronger error-correcting capabilities, theoretically offering potential advantages such as larger minimum distances, greater flexibility in component code selection for check nodes, lower error rates under short-to-medium block lengths, faster decoding convergence, and reduced error floors. Moreover, they can achieve higher NCG at the same code rate compared to RS codes and reduce encoding complexity and memory overhead through structured designs (e.g., QC-LDPC), making them suitable for hardware implementation. Their application potentials are primarily focused on scenarios with extremely high reliability and low latency requirements, such as beyond-100G optical transmission systems (e.g., 1.6T), visible light communication (VLC) systems, massive machine-type communications (mMTCs), and integrated optical-wireless networks (e.g., 5G/6G fronthaul and midhaul). These scenarios require resilience against nonlinear noise and complex channel impairments, where the strong error correction capability and flexibility of GLDPC codes can significantly enhance system robustness. At the present stage, the application research of GLDPC codes in optical communications is still in the exploratory stage, which have not yet been directly adopted in mainstream optical communication standards (e.g., ITU-T or OIF specifications). However, as optical communications evolve toward single-wavelength 800G/1.6T and leverage technologies like C+L band expansion, GLDPC codes are expected to be one of the key technologies for beyond-400G standards.

**Figure 1:**
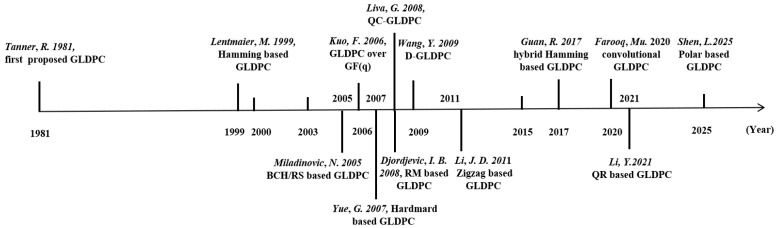
The research progress of GLDPC codes [1,2,3,4,5,6,9,10,11,12,13,14,18].

Figure 1 illustrates the development timeline of GLDPC codes. Tanner first proposed that check nodes need not be limited to SPC nodes but could instead adopt any generalized parity check structure, thereby introducing the concept of GLDPC codes. However, Tanner did not provide concrete GLDPC code constructions [1]. In 1999, Lentmaier et al. systematically expanded LDPC codes by employing Hamming codes as component codes for check nodes, marking the beginning of structured GLDPC research [2]. Since then, various GLDPC architectures have emerged, including non-binary GLDPC codes [3], quasi-cyclic GLDPC codes [4], doubly generalized GLDPC codes [5], and convolutional GLDPC codes [6,7,8]. For component codes, scholars have explored diverse candidates such as BCH codes [9], Reed–Solomon (RS) codes [9], Hadamard codes [10], Reed–Muller (RM) codes [11], Zigzag codes [12], Hamming codes [2,13], and Quadratic Residue (QR) codes [14]—wherein Hamming codes [15,16,17], BCH codes, and RM codes [11] have become common choices in optical communication systems due to their good balance between bit error performance and implementation complexity. In 2025, authors in [18] proposed a novel GLDPC code using polar codes as component codes (termed GLDPC-PC codes), where soft information from polar components is efficiently extracted from the soft-output successive cancellation list (SO-SCL) decoder. GLDPC-PC codes exhibit outstanding block error rate (BLER) performance, particularly in ultralow error regimes, aligning with 6G communication requirements for higher data rates and ultrareliable transmission.

Particularly, the generalized low-density parity check convolutional code (GLDPC–CC) is a class of GLDPC codes. The convolution structure brings about better waterfall performance, which makes GLDPC–CC a very promising solution for optical communication. In [16], a kind of GLDPC–CC is proposed with Hamming (15,11) code as the component code. The layered decoding algorithm is used in the decoding implementation, where the SPC nodes adopt the revised min-sum algorithm, and GC nodes use the Bahl–Cocke–Jelinek–Raviv (BCJR) algorithm [17]. However, the authors found that the proposed GLDPC–CC has no obvious advantages over LDPC convolutional code (LDPC–CC) [19] in terms of error correction performance, delay, and power consumption.

In this paper, we focus on the research of GLDPC–CC codes that are suitable for high-reliability and low-latency optical communication scenarios. In order to further improve the error correction performance while reduce the decoding delay and power consumption, we conducted a series of studies on various aspects of GLDPC–CCs, including the code construction, decoding algorithm, and low-power strategies. The contributions of this paper can be summarized as the following two aspects:

(1) Firstly, we re-design GLDPC–CCs from the perspective of code structure, aiming to enhance the error-correction performance.
  - Considering that the existing GLDPC–CCs adopt Hamming codes as the component code whose error-correction performance is weak and the construction method is not flexible, a novel doping method of replacing multiple SPC nodes with one GC node is proposed, in which the joint optimization strategy of GC node types and GC node ratios is adopted. This method enables the construction of BCH codes of various lengths as component codes, and also allows for the flexible construction of GLDPC–CCs with different code rates.
  - Moreover, the influence of GC node distribution on the coupling structure is discussed, which indicates that the nonuniform intra-layer GC node distribution can lead to better error correction performance compared to the uniform GC node distribution.
(2) Secondly, in terms of the decoding algorithm aspect, considering that the BCJR algorithm is only applicable to simple code types such as Hamming codes, for the BCH code family with longer code lengths and stronger error correction capabilities, the decoding complexity of the BCJR algorithm will sharply increase and will no longer be applicable. Therefore, we develop a decoding algorithm and its optimized versions suitable for GLDPC–CCs with GC nodes of BCH or RS codes.
  - A hybrid layered normalized minimum sum (HLNMS) decoding scheme of GLDPC–CCs is proposed in this paper, where the layered normalized minimum sum (LNMS) decoding is adopted for the single parity check (SPC) node, and the Chase–Pyndiah decoding is adopted for the generalized check (GC) node, which has lower decoding delay and storage complexity.
  - Moreover, in order to further improve the error correction performance of proposed HLNMS algorithm, an optimized adaptive weighting factor of the Chase–Pyndiah algorithm for GC nodes is proposed based on the analysis of the decoding convergence characteristics of GC nodes and SPC nodes.
  - On the other hand, in terms of low-power consumption strategies, a decoding early stopping strategy is also proposed for the HLNMS algorithm to reduce the decoding complexity while maintaining comparable error correction performance.

Among the above mentioned works, the proposed HLNMS algorithm is an optimized scheme of the decoding algorithm of [16,17], where the Chase–Pyndiah decoding for GC nodes of BCH code is different from the BCJR decoding for GC nodes of Hamming code in [16,17]. The other proposed encoding and decoding schemes are all original.

The remainder of this paper is organized as follows. The background of generalized LDPC codes is briefly introduced in Section 2. Specific descriptions of the proposed encoding and decoding schemes are provided in Section 3 and Section 4. The numerical analyses and simulation results are presented in Section 5, followed by the conclusions in Section 6.

## 2. Preliminaries

In this section, we briefly introduce the basic concepts, characteristics, and common construction methods of GLDPC codes, as well as the prior-art construction method of GLDPC–CCs.

### 2.1. Basic Definitions of GLDPC Codes

GLDPC codes were first proposed by Tanner in 1981 [1]. Unlike traditional LDPC codes that use single parity check (SPC) codes as check nodes, which can only detect one error, GLDPC codes employ component codes with certain error correction capabilities as check nodes. From the perspective of composition structure, GLDPC codes are an extension of standard LDPC codes. Therefore, they are also a kind of linear block code whose parity check matrix satisfies the sparse property and conforms to all the corresponding characteristics of linear block codes. That is, GLDPC codes have the generator matrix G and the corresponding parity check matrix H, which satisfy $G\cdot H^{T}=0$. In the parity check matrix of a standard LDPC code, each row corresponds to a parity check constraint equation, and this parity check constraint conforms to the parity check relationship of an SPC code; each column represents the information of a corresponding parity check variable. Compared with the parity check matrix of a standard LDPC code, the parity check matrix of a GLDPC code shows no difference in form, and the two can even be used interchangeably. However, there is an essential difference in their physical meanings. That is, the parity check constraints that a GLDPC code needs to satisfy can be selected from many other codes besides SPC codes, such as Hamming codes, BCH codes, etc. Therefore, in its parity check equations, it no longer satisfies the parity check relationship of one SPC per row, but instead, multiple rows represent one parity check relationship, jointly satisfying the constraint conditions of the corresponding parity check component codes, which are called generalized parity check (GC) nodes.

Let *R* be the code rate of GLDPC codes, and let the variable node degree of GLDPC codes be $d_{v}$, and the check node degree be *n*. The component code is a linear block code C(n,k) with a code rate of r=k/n. Then the code rate *R* must satisfy the following relationship:

(1) $$R\geq 1-\left(1-r\right)d_{v}$$

Tanner derived the relationship between the lower bound of the minimum Hamming distance and the parameters of the component code in [1]. Let *d* be the minimum Hamming distance of the component code, *D* be the minimum Hamming distance of GLDPC, the degree of the bit node be $d_{v}$, and the circumference of the Tanner graph be *g*. Then, the following relationship holds:

(2) $$D\geq \left\{\begin{array}{cc} d\frac{{\left[\left(d-1\right)\left(d_{v}-1\right)\right]}^{\frac{g-2}{4}}-1}{\left(d-1\right)\left(d_{v}-1\right)-1}+\frac{d}{d_{v}}{\left[\left(d-1\right)\left(d_{v}-1\right)\right]}^{\frac{g-2}{4}}, & \;g/2\;\;\mathrm{is}\;\mathrm{odd};\;\\ d\frac{{\left[\left(d-1\right)\left(d_{v}-1\right)\right]}^{\frac{g}{4}}-1}{\left(d-1\right)\left(d_{v}-1\right)-1}, & g/2\;\mathrm{is}\;\mathrm{even}.\; \end{array}\right.$$

Suppose the code length of the component code is *n*, and the code length of the GLDPC code is *N*, then there is the following relationship:

(3) $$N\geq \left\{\begin{array}{cc} n\frac{{\left[\left(n-1\right)\left(d_{v}-1\right)\right]}^{\frac{g-2}{4}}-1}{\left(n-1\right)\left(d_{v}-1\right)-1}+\frac{n}{d_{v}}{\left[\left(n-1\right)\left(d_{v}-1\right)\right]}^{\frac{g-2}{4}}, & g/2\;\;\mathrm{is}\;\mathrm{odd};\;\\ n\frac{{\left[\left(n-1\right)\left(d_{v}-1\right)\right]}^{\frac{g}{4}}-1}{\left(n-1\right)\left(d_{v}-1\right)-1}, & g/2\;\mathrm{is}\;\mathrm{even}.\; \end{array}\right.$$

Lentmaier proved in [2] that the minimum Hamming distance of GLDPC linearly increases with the code length, and provided the lower bound formula as follows:

(4) $$D\geq N\delta \left(d_{v},\;n\right)+o\left(N\right),$$

where $\delta (d_{v},\;n)$ is a coefficient that is related to the variable node degree $d_{v}$ and the code length *n* of the component code.

### 2.2. Construction Methods of GLDPC Codes

In the terms of the parity check matrix construction of GLDPC codes, there are two classic construction methods.

The first construction method proposed by Boutrous in [20] is to extend the structure of Gallager’s LDPC codes, by replacing each parity check equation of the (N,K) LDPC codes with a small parity check matrix of the (n,k) linear component code. Firstly, a parity check matrix H of regular LDPC codes is constructed by using the Gallager’s construction method. Secondly, each row of the H matrix is replaced with the parity check matrix $H_{0}$ of the (n,k) component code, thereby generating a new parity check matrix $H_{1}$. Suppose the parity check matrix $H_{g}$ of GLDPC code has *J* submatrices. Then set $H_{1}$ as its first submatrix, and the other J−1 submatrices are generated by column permutations of $H_{1}$.

The second construction method proposed by Lentmaier in [2] is basically the same as that of Boutrous’s method, which also extends the initial LDPC matrix H by using the parity check matrix $H_{0}$ of (n,k) component codes. The difference is that the non-zero elements in each row of the parity check matrix H are permutations with *n* different column vectors in $H_{0}$ and the zero elements in each row of H matrix are permutations with a completely zero column vector of length n−k to obtain the parity check matrix $H_{g}$ of GLDPC codes.

### 2.3. Construction Method of GLDPC–CCs

**Figure 2:**
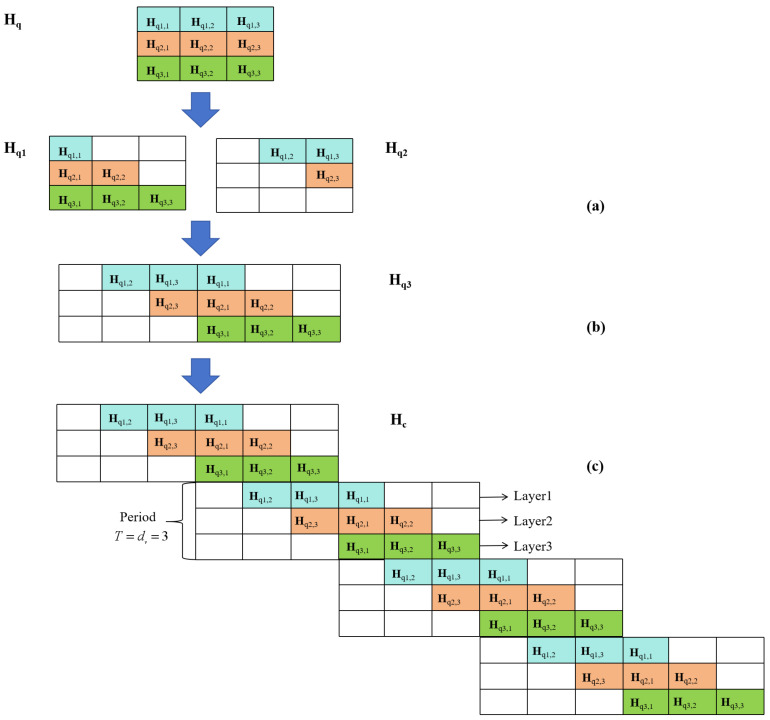
Construction process of the parity check matrix of GLDPC–CCs: (**a**) Cutting operation; (**b**) Splicing operation; (**c**) Copying and pasting operations.

The GLDPC–CC in [16] is a generalized extension of the quasi-cyclic (QC) LDPC–CC. The adoption of a quasi-cyclic structure is to enhance the hardware feasibility. The specific construction process of the parity check matrix of GLDPC–CCs is shown in Figure 2, where $H_{q}$ is a quasi-cyclic matrix expanded by the base matrix $H_{b}$ with a row weight of $d_{c}$ and a column weight of $d_{v}$ according to the cyclic shift factor matrix $H_{shift}$ and the expansion factor *Z*. The base matrix $H_{b}$ is a full one matrix.

As shown in Figure 2a, each row of the $H_{q}$ matrix is evenly divided into $d_{v}$ sub-matrices, denoted as $H_{q_{i,j}}$ (i, j = 1, 2, …, $d_{v}$). Each $H_{q_{i,j}}$ contains dc/dv cyclic blocks. Note that $d_{v}$ is set as 3 and $d_{c}$ is set as 15 in this paper. This partitioning strategy is used for the subsequent convolution operations of $H_{q}$. In Figure 2a, the $H_{q}$ matrix is cut along the diagonal to obtain the upper right part $H_{q2}$ and the lower left part $H_{q1}$. Subsequently, $H_{q2}$ and $H_{q1}$ are spliced left and right to obtain $H_{q3}$ as shown in Figure 2b. The cutting and splicing operations are carried out to prepare for the subsequent convolution operations. As shown in Figure 2c, by repeatedly copying and pasting $H_{q3}$, the parity check matrix $H_{c}$ of the QC–LDPC–CC code can be formed. The period of the QC–LDPC–CC codes is $T=d_{v}=3$. Finally, by replacing certain rows of $H_{c}$ with the parity check matrix $H_{0}$ of the component code, the parity check matrix $H_{g}$ of GLDPC–CCs can be easily constructed.

## 3. Optimized Encoding Scheme of GLDPC–CCs

This section focuses on the optimized encoding schemes of GLDPC–CCs. Firstly, the matching selection of generalized code types and the generalized check node ratios is discussed. Then, the effect of the GC nodes distribution on the degree distribution characteristics of GLDPC–CCs is analyzed.

### 3.1. Joint Optimization of GC Node Proportion and GC Component Code Type

In the encoding scheme described in [16], one single SPC node is replace with a GC node of Hamming (15, 11) code. This encoding scheme results in a uniform and fixed GC component code type, which prevents any further improvement on error correction performance. Accordingly, an optimized doping method of replacing multiple SPC nodes with a single GC node. Accordingly, with the same code rate, different combinations of component code types and GC nodes proportion can be obtained, and the optimal combination with the best error correction performance can be selected.

**Figure 3:**
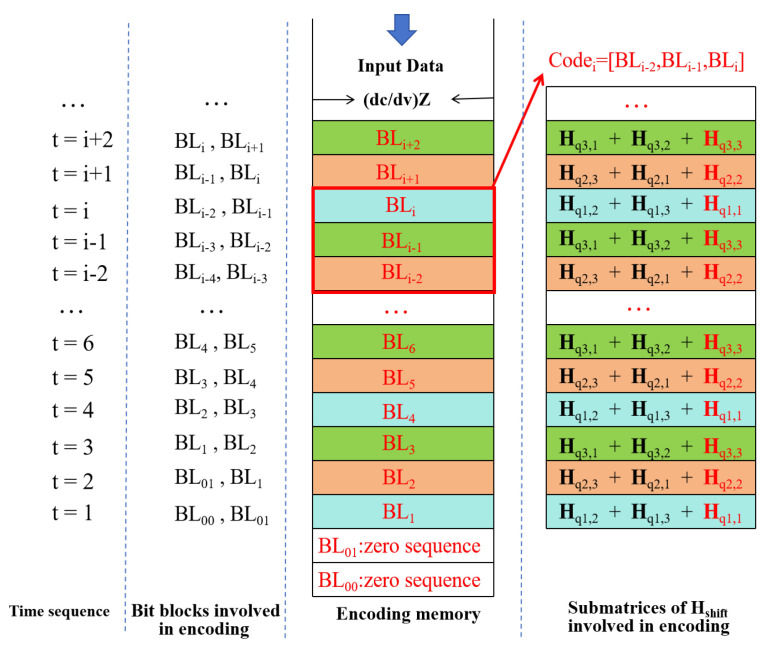
Encoding process of GLDPC–CCs.

Figure 3 illustrates the specific encoding process of GLDPC–CCs. As shown in Figure 3, the length of each row of encoding memory is (dc/dv)Z, where *Z* is the cyclic shift expansion factor. All $d_{v}$ rows of the encoding memory are coupled together to generate a long code of dcZ bits. The specific encoding process is as follows.

Step 1: Initialization. Load the codewords in the (*i*−2)-th and the (*i*−1)-th row of encoding memory as the first 2(dc/dv)Z bits of the codeword ${\mathrm{Code}}_{i}$, and initialize the last (dc/dv)Z bits to be zeros, that is, ${\mathrm{Code}}_{i}=[BL_{i-2},\;BL_{i-1}$, zeros(1, (dc/dv)Z)]. Especially, when i=1, ${\mathrm{Code}}_{i}=\left[BL_{00},\;BL_{01},zeros\left(1,\;\left(dc/dv\right)Z\right)\right]$; and when i=2, ${\mathrm{Code}}_{i}=\left[BL_{01},\;BL_{i-1},\;zeros\left(1,\;\left(dc/dv\right)Z\right)\right]$. Here, $BL_{00}$ and $BL_{01}$ are all-zero sequences.

Step 2: Determine the sub-matrix of $H_{shift}$ involved in encoding. For the *i*-th row of encoding memory, the *k*-th row of the cyclic shift matrix $H_{shift}\left(K,\;:\right)$ is involved in encoding, where $K=mod(i,\;d_{v})$.

Step 3: Encode the codeword ${\mathrm{Code}}_{i}$ at the *i*-th row of the encoding memory.

(1) SPC node encoding

For the j-th $\left(d_{c}-1,\;dc\right)$ SPC node, the first 2(dc/dv) bits are firstly searched according to $H_{shift}\left(K,\;1:2\left(dc/dv\right)\right)$, and dc−2(dc/dv)−1 new information bits need to be randomly generated. Then one check bit is generated, and through the addition operation in GF(2), similarly the newly generated dc−2(dc/dv)−1 information bits and one check bits are placed at the corresponding positions of ${\mathrm{Code}}_{i}$ according to $H_{shift}\left(K,\;2\left(dc/dv\right)+1:d_{c}\right)$.

(2) GC node encoding by replacing *W* (W≥1) SPC nodes with a single GC node.

For j∈[1,Z], replace the *W* SPC rows with a GC node every W/P rows. First, search for the corresponding first 2(dc/dv) bits of the *W* rows of SPC nodes according to $H_{shift}\left(K,\;1:2\left(dc/dv\right)\right)$, and merge them sequentially into the first 2(dc/dv)W information bits. Then, generate k−2(dc/dv)W new information bits, and add *J* zeros (where *J* is the number of truncated bits) at the beginning of the information sequence, merging them into k+J information bits. Next, encode n−k check bits using BCH (n+J, k+J) code. Finally, place the newly generated k−2(dc/dv)W information bits and n−k check bits at the corresponding positions of $code_{i}$ according to $H_{shift}\left(K,\;2\left(dc/dv\right)+1:dc\right)$. Table 1 provides different combinations of component code types and the GC nodes proportion with the same code rate of 0.775, where the code rate can be calculated as follows:

(5) $$R=1-\frac{d_{v}}{d_{c}}\cdot \left[1+\left(\frac{n-k}{W}-1\right)\cdot P\right],$$

where *W* is the number of SPC nodes to be replaced with a GC node; *P* is the GC node proportion; $d_{v}$ and $d_{c}$ are the column weight and row weight of base matrix $H_{b}$, respectively; and *n* and *k* are the codeword length and information length of the BCH code, respectively.

In particular, the component code type BCH (60,53) in Table 1 is an expanded-truncated BCH code with one extended parity check bit and one more truncated bit compared with the BCH (60,54) code type. Moreover, in order to keep the same code rate of 0.775, the GC node proportion is reduced to 1/6. The purpose of adding this extended parity check bit is aimed at obtaining better error correction performance.

### 3.2. Analysis of the GC Node Distribution

In this section, the effects of intra-layer GC node distribution on the inter-layer GC node distribution is discussed. The inter-layer GC node distribution refers to how many GC nodes a certain bit is checked by. As seen in Figure 2c, the coupling length of the considered GLDPC–CCs is $d_{v}$, which means that each bit is checked by $d_{v}$ check nodes. Among the $d_{v}$ check nodes, the higher the proportion of GC nodes, the greater the probability that the erroneous bit will be corrected. Therefore, we statistically analyzed various situations which include two or more GC nodes among all $d_{v}$ check nodes corresponding to the coding structure of Figure 2c. As shown in Table 2, there are six types in total, which include two more GC nodes. Take Type 1 as an example. It indicates that when one bit node is encoded using the first and second layers of cyclic shift factor matrix $H_{shift}$, respectively, it is checked by the GC node. While this bit node is encoded by the third layer of $H_{shift}$, it is checked by the SPC node. Therefore, in the case of Type 1, one bit node is checked by a total of two GC nodes and one SPC node.

Given a certain cyclic shift factor matrix $H_{shift}$ and the cyclic shift expansion factor *Z*, different intra-layer GC node distributions would greatly affect the proportions of the inter-layer distribution types listed in Table 2. Here we compared two cases: the uniform distribution and the nonuniform distribution. For the uniform intra-layer distribution, the codeword is encoded by uniformly spaced GC and SPC nodes, while for the nonuniform distribution, the codeword is firstly encoded by GC nodes, and then by SPC nodes.

Take the GC node of BCH (60,53) code as an example, where the code length of GLDPC–CCs is set as 19800 bit. The numbers of bit nodes corresponding to the six types of inter-layer GC node distribution are denoted as $N_{GC}=\left[N_{1},\;N_{2},\;N_{3},\;N_{4},\;N_{5},\;N_{6}\right]$. For the uniform intra-layer distribution, $N_{GC}=\left[165,\;0,\;110,\;0,\;110,\;0\right]$, which has no Type 2, 4, and 6 with 3 GC nodes. For the nonuniform intra-layer distribution, $N_{GC}=\left[522,\;151,\;1215,\;95,\;288,\;0\right]$, which has more types with three GC nodes and is approximately six times the uniform distribution in total. That is to say, the nonuniform intra-layer GC node distribution can lead to more inter-layer GC node distributions which have two more GC nodes.

## 4. Decoding Schemes of GLDPC–CCs

This section discusses the decoding scheme of GLDPC–CCs, including the parallel hybrid decoding architecture, the hybrid layered normalized minimum sum (HLNMS) decoding scheme of GLDPC–CCs, and two kinds of optimized HLNMS decoding schemes.

### 4.1. Parallel Hybrid Decoding Architecture of GLDPC–CCs

**Figure 4:**
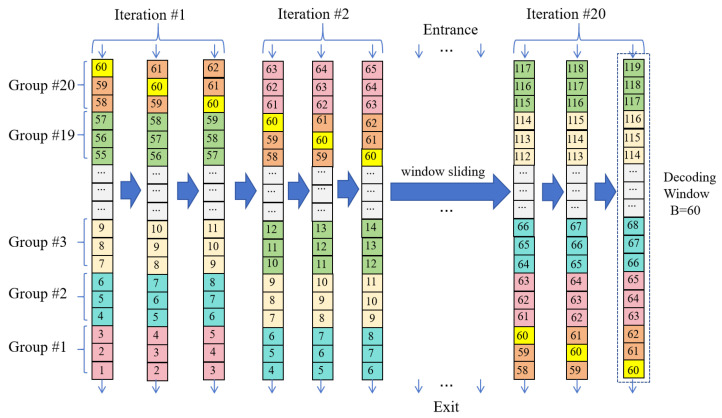
Parallel decoding architecture diagram of GLDPC–CCs.

The parallel hybrid decoding architecture of GLDPC–CCs is shown in Figure 4. The decoding window contains *B* blocks (B=60 in Figure 4), and every three blocks form one group (represented with different color blocks), i.e., a complete codeword of dcZ bits, which are. Thus, there are 20 codewords in a decoding window. The mutual information (MI) of *i*-th codeword (i∈[1,20]) is loaded as $L_{i}=\left[L_{3i-2},\;L_{3i-1},\;L_{3i}\right]$. These groups can be simultaneously decoded, which can significantly improve the decoding efficiency and throughput. In the decoding window, the cyclic shift sub-matrix corresponding to each group is consistent. For ease of processing, we can uniformly represent it using the corresponding sub-matrix of the third row or the B-th row, and denote it as $H_{shift}\left(K,\;:\right)$, where K=1,2,3. After decoding is completed, the block at the bottom of the decoding window is output, and a new block is entered from the top of the decoding window. As shown by the arrows in Figure 4, this operation can be understood as the decoding window sliding as, thereby initiating a new round of decoding. Through this continuous window sliding mechanism, continuous decoding of the data is achieved. When the window slides three times, one block will be decoded by all the three sub-matrices of $H_{shift}$, and thus one decoding iteration is complete.

### 4.2. Hybrid Layered Normalized Minimum Sum (HLNMS) Decoding of GLDPC–CCs

The proposed hybrid layered normalized minimum sum (HLNMS) decoding algorithm consists of two parts. The SPC node adopts the layered normalized minimum sum (LNMS) decoding algorithm, and the GC node adopts the Chase–Pyndiah decoding algorithm [21,22]. These two types of check nodes carry out the fusion of mutual information under the framework of LNMS decoding, which can be described as the following:

(6) $$\begin{array}{c} L^{\left(l,\;k\right)}\left(q_{j,\;i}\right)=L^{\left(l,\;k-1\right)}\left(Q_{j}\right)-L^{\left(l-1,\;k\right)}\left(r_{i,\;j}\right),\\ L^{\left(l,\;k\right)}\left(r_{i,\;j}\right)=\alpha \cdot \prod_{j\in N(i)/j}\mathrm{sgn}\left[L^{\left(l,\;k\right)}\left(q_{j,\;i}\right)\right]\cdot \min_{j\in N(i)/j}\left|L^{\left(l,\;k\right)}\left(q_{j,\;i}\right)\right|,\\ L^{\left(l,\;k\right)}\left(Q_{j}\right)=L^{\left(l,\;k\right)}\left(q_{j,\;i}\right)+L^{\left(l,\;k\right)}\left(r_{i,\;j}\right), \end{array}$$

where *l* is the number of decoding iterations; *k* is the number of decoding layers; $L^{\left(l,\;k\right)}\left(q_{j,\;i}\right)$ is the mutual information of variable nodes; $L^{\left(l,\;k\right)}\left(r_{i,\;j}\right)$ is to verify the mutual information of check nodes; $L^{\left(l,\;k\right)}\left(Q_{j}\right)$ is the posterior information of the variable node; and α is the normalization factor, which is set to 0.75. The specific decoding process is as follows.

Step 1: Initialization. The initial information of $L^{\left(l,\;k\right)}\left(Q_{j}\right)$ is set as the channel information $L_{ch}$; the initial information of $L^{\left(l,\;k\right)}\left(r_{i,\;j}\right)$ is 0.

Step 2: If the *j*-th row (j∈[1,Z]) of the *i*-th codeword (i∈[1,20]) is a SPC node, search the corresponding LLRs according to $H_{shift}\left(K,\;:\right)$, then update them with (6).

Step 3: If the *j*-th row (j∈[1,Z]) of *i*-th codeword (i∈[1,20]) is a GC node, search the corresponding MI according to $H_{shift}\left(K,\;:\right)$, then decode them with the Chase–Pyndiah decoder. The input of the decoder is the updated $L^{\left(l,\;k\right)}\left(q_{j,\;i}\right)$, and the output is the updated $L^{\left(l,\;k\right)}\left(r_{i,\;j}\right)$.

Step 4: When the mutual information of all *Z* nodes has been updated, aggregate the MI of GC nodes and SPC nodes, and update the posterior probability $L^{\left(l,\;k\right)}\left(Q_{j}\right)$.

Step 5: After updating the posterior information $L^{\left(l,\;k\right)}\left(Q_{j}\right)$ of all blocks, directly perform hard decision on $L^{\left(l,\;k\right)}\left(Q_{j}\right)\left(1,\;:\right)$ and output the result from the bottom of the decoding window. Meanwhile, the MI of a new block enters from the top of the decoding window. Then, return to Step 2 to start a new round of decoding.

### 4.3. Complexity Analysis of HLNMS Decoding Scheme

In this subsection, we provide a quantitative analysis of the computational complexity, decoding latency, throughput, and memory usage for the proposed HLNMS decoding scheme. The parameters involved are hereby restated as follows:

- *Z*: Cyclic shift expansion factor;
- $d_{c}$: Row weight of the base matrix;
- $d_{v}$: Column weight of the base matrix;
- *P*: Proportion of GC nodes in the check nodes;
- *W*: Number of SPC nodes to be replaced together with a GC node;
- *n*: Length of the component code used in a GC node;
- *t*: Number of test patterns in the Chase–Pyndiah algorithm for GC nodes;
- Maxiter: Maximum number of decoding iterations;
- *B*: Decoding window size.

(1) Computational Complexity

The total computational complexity for decoding one codeword is

(7) $$C_{\mathrm{total}\;}=Maxiter\cdot d_{v}\cdot Z\left[\left(1-P\right)\cdot O\left(d_{c}\right)+P\cdot W\cdot O\left(2^{t}\cdot n\right)\right],$$

where $O\left(d_{c}\right)$ is the complexity per SPC node (LNMS update), and $O\left(2^{t}\cdot n\right)$ is the complexity per GC node (Chase–Pyndiah decoding), which is the dominant factor of Equation (7).

(2) Decoding latency

The end-to-end latency for decoding one codeword is

(8) $$D=Maxiter\cdot d_{v}\cdot \tau_{\mathrm{GC}},\;\tau_{\mathrm{GC}}\propto 2^{t}\cdot n.$$

The decoding latency scales linearly with Maxiter, $d_{v}$ and $\tau_{\mathrm{GC}}$, where $Maxiter\cdot d_{v}$ is the total window slides, and $\tau_{\mathrm{GC}}$ is the processing delay per GC node. Note that $\tau_{\mathrm{GC}}$ is a hardware-dependent constant, the actual value of which depends on implementation (ASIC/FPGA parallelism, quantization, etc.).

(3) Throughput

The throughput (decoded bits per unit time) is

(9) $$\;\mathrm{Throughput}\;=\frac{d_{c}\cdot Z}{d_{v}\cdot \tau_{\mathrm{slide}\;}}\;\;\mathrm{where}\;\;\tau_{\mathrm{slide}\;}\propto \tau_{\mathrm{GC}},$$

where $d_{c}\cdot Z$ is the codeword length (bits), $\tau_{\mathrm{slide}\;}$ is the time per window slide (driven by $\tau_{\mathrm{GC}})$, and $d_{v}\cdot \tau_{\mathrm{slide}\;}$ is the time to process one full iteration.

(4) Memory usage

The total memory required for the sliding-window decoder is as follows:

(10) $$M_{\mathrm{total}}=B\cdot \frac{d_{c}}{d_{v}}Z\cdot \left(m_{v}+m_{c}\right)+P\cdot W\cdot B\cdot Z\cdot O\left(2^{t}\cdot n\right).$$

The first term of Equation (10) is the block memory, where $m_{v}$ and $m_{c}$ are the memory per variable node and memory per check node, respectively. The second term is the GC node-specific memory, which stores test patterns and metrics for Chase–Pyndiah decoding.

To sum up, due to the fully parallel decoding structure, the computational complexity, decoding delay, and throughput of the proposed HLNMS decoding algorithm mainly depend on the decoding algorithm of one GC node. Furthermore, since the Chase–Pyndiah algorithm adopted by the GC nodes has the characteristics of simple calculation, fast implementation, and excellent performance; thus, the proposed HLNMS decoding algorithm has the features of low computational complexity, small decoding latency, and high throughput, which is very conducive to hardware implementation.

### 4.4. Optimized HLNMS Decoding Schemes for GLDPC–CCs

In this section, two kinds of optimization schemes are proposed for the HLNMS decoding algorithm described in Section 4.2. The first one is aimed to improve the decoding performance, and the another one is used to reduce the decoding complexity while maintaining the error correction performance.

Scheme I: Adaptive weighting factor for GC node decoding

As shown in Figure 4, a code block (such as block #60) needs to go through 60 times of window sliding, i.e., 20 decoding iterations, from entering to sliding out of the decoding window. Its mutual information becomes more reliable during the iterative process. Therefore, in the same decoding window, the reliabilities of 60 blocks are different, which gradually increase from the entrance to the exit of the decoding window.

Based on this observation, we proposed an adaptive weighting factor strategy named as Scheme I, which assigns different weighting factors for GC nodes with different levels of reliability. The reliability of mutual information can be simply divided based on the position of GC nodes within the decoding window. It can also be understood as the same GC node in a code block being assigned different weighting factors for the different decoding iterations. The specific weighting factor values are derived empirically based on simulations. In order to make the hardware implementation convenient, these weighting factor values can be directly converted into binary form as follows:

(11) $$\alpha_{GC}=\left\{\begin{array}{cc} 0.75, & 1\leq i<0.2B\\ 0.71825, & 0.2B\leq i<0.4B\\ 0.6875, & 0.4B\leq i<0.6B\\ 0.65625, & 0.6B\leq i<0.8B\\ 0.5625, & 0.8B\leq i\leq B \end{array}\right.,$$

where *B* is the window size, and *i* is the block index of the decoding window. As shown in (11), the blocks close to the exit are assigned to larger weighting factors.

Scheme II: Early stopping strategy for GC node decoding

It is found that the mutual information of the GC node increases significantly faster with the decoding iteration than that of the SPC node, and thus the GC nodes could converge more quickly. Accordingly, a low-power decoding strategy is proposed as follows.

If the minimum amplitude of MI is greater than a certain threshold Th, it indicates that the reliability of this GC node is already very high or has converged. Decoding this GC node is no longer necessary to reduce the decoding complexity and decoding power consumption. If the threshold is selected appropriately, the decoding complexity/power consumption can be reduced on the premise of ensuring no loss of decoding performance. The selection of the threshold can be obtained through the offline analysis of the decoding convergence characteristics of GC nodes and SPC nodes, which is detailed as follows.

Step 1: Analyze offline the trend of the minimum MI amplitude of GC and SPC nodes in the high SNR range as the decoding iterations, and find the maximum value Th_0_ of the minimum MI amplitude of GC node when it converges.

Step 2: The initial value of Th for the minimum MI amplitude is set as Th_0_.

Step 3: Before decoding at the GC node, a judgment is made: if the minimum MI amplitude of the GC node is greater than Th, then the decoding of this GC node will not be carried out; otherwise, the decoding will continue.

Step 4: After the decoding, if the post_FEC BER exceeds the preset tolerance value $BER_{Gap}$ compared to the post_FEC BER without implementing the low-power control, then increase Th and return to Step 3; otherwise, terminate the search and output Th.

Note that the tolerance parameter $BER_{Gap}$ is a parameter used to balance the BER performance and the power consumption. The smaller the $BER_{Gap}$, the less that BER performance deteriorates and the less the power consumption is reduced.

## 5. Simulation and Analysis

In this section, we present a performance comparison and analysis of different combinations of GC node types and proportions, the convergence analysis of GC nodes and SPC nodes, the performance analysis of the two optimized HLNMS decoding algorithms, and finally, we will conduct a performance comparison between the proposed algorithm and the algorithm in the related literature.

Given that this work focuses on studying GLDPC codes that are suitable for optical communication scenarios that require simultaneously meeting the requirements of high reliability and low processing delay, we select a set of parameter configurations with a code rate lower than 0.8 and a code length of approximately 20,000 bits in the simulations. Regarding the setting of a code length of approximately 20,000 bits, we refer to references [23,24,25]; regarding the settings of the code rate and overhead, we refer to references [16,17]; in terms of code type selection, reference is made to [11]. The specific coding parameters are set as follows. The circular shift expansion factor is set as Z=1320, the block length is $d_{c}Z=19,800$ bits, and the code rate is 0.775. The normalization factor of LNMS decoding for SPC nodes is α=0.75. Note that the specific parameter settings of the proposed algorithm are based on the actual engineering of optical communication systems. For the sake of generality, we present the simulation results in two ways. One way is to adopt the bit error rate before error correction (pre−FEC BER) as the horizontal axis, and the other way is to use the signal-to-noise ratio (SNR) as the horizontal coordinates.

### 5.1. Performance Comparison of Combinations of Component Code Types and GC Node Proportions

**Figure 5:**
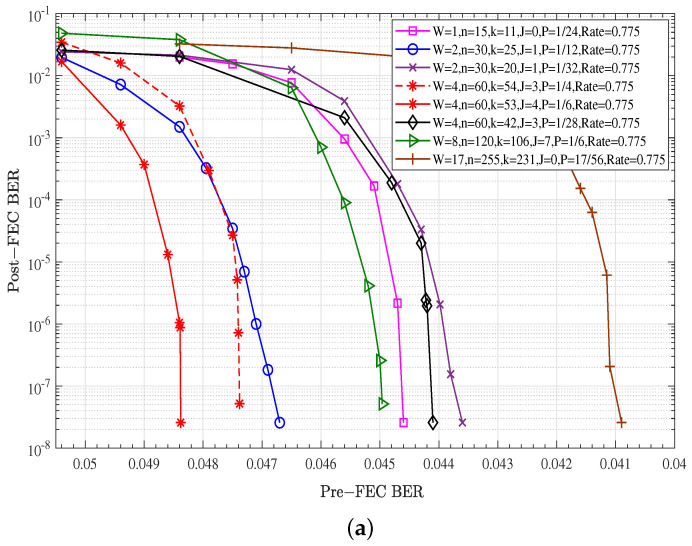

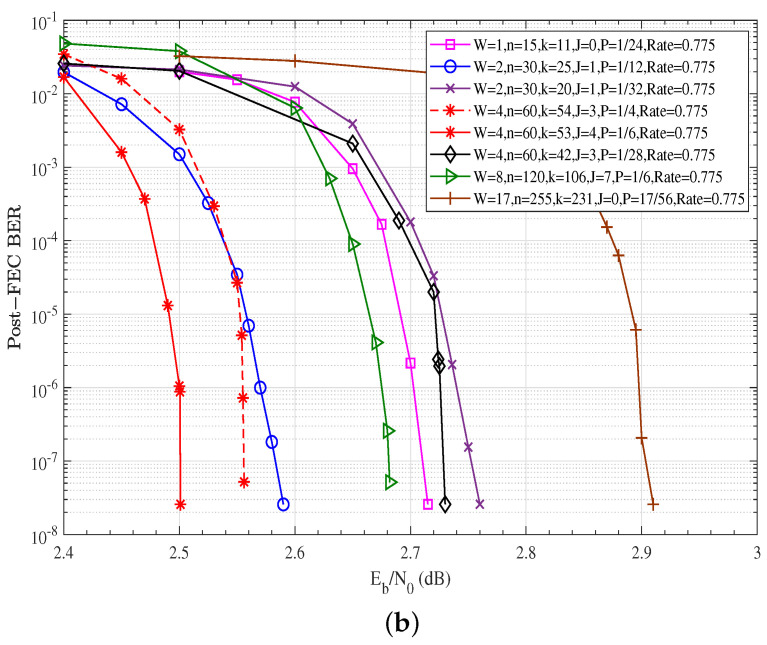
Performance comparison of combinations of GC node types and GC node proportions: $\alpha_{GC}=1$, α=0.75, B=60, Maxiter=20: (**a**) Post-FEC BER versus Pre-FEC BER; (**b**) Post-FEC BER versus SNR.

Figure 5 presents the performance comparisons of all combinations of code types and GC node proportions listed in Table 1 with a fixed code rate of 0.775. The fixed weighting factor of Chase–Pyndiah decoding for GC nodes is also $\alpha_{GC}=1$. The x-axis of Figure 5a is the bit error rate before error correction (Pre-FEC BER), and the Y-axis is the bit error rate after error correction (Post-FEC BER). The x-axis of Figure 5b is the signal-to-noise ratio (SNR).

Impact of *W* on performance: *W* is the number of SPC nodes to be replaced with a GC node. When the code rate remains at 0.775, as the value of *W* gradually increases to 4, the performance of GLDPC–CCs continuously improves. However, as the value of *W* further increases, the performance shows a downward trend. Therefore, the combination with W=4 has the best performance. This indicates that there is an optimal range for the value of *W*, within which the performance advantages can be fully exploited.

Impact of the GC node proportion *P* on performance: for the combinations with the same parameters of *W* and code rate, it can be found that the larger the GC node proportion *P*, the better the error performance.

Impact of the extended parity check node on performance: As seen in the two red curves in Figure 5, the BCH (60,53) code (red solid line) is constructed based on BCH (60,54) code (red dashed line) by adding one parity check node, adding one bit of truncated bit, and also decreasing the GC node proportion *P* to keep the same code rate. The results show that the performance improvement brought by this optimization strategy is quite significant. To sum up, we obtained a set of optimal parameters with best error correction performance, i.e., W=4, P=1/6, n=60, k=53, J=4, R=0.775. Therefore, the subsequent analysis and optimization encoding and decoding schemes are based on these settings.

### 5.2. Analysis of the Impact of Window Size on Decoding Performance

**Figure 6:**
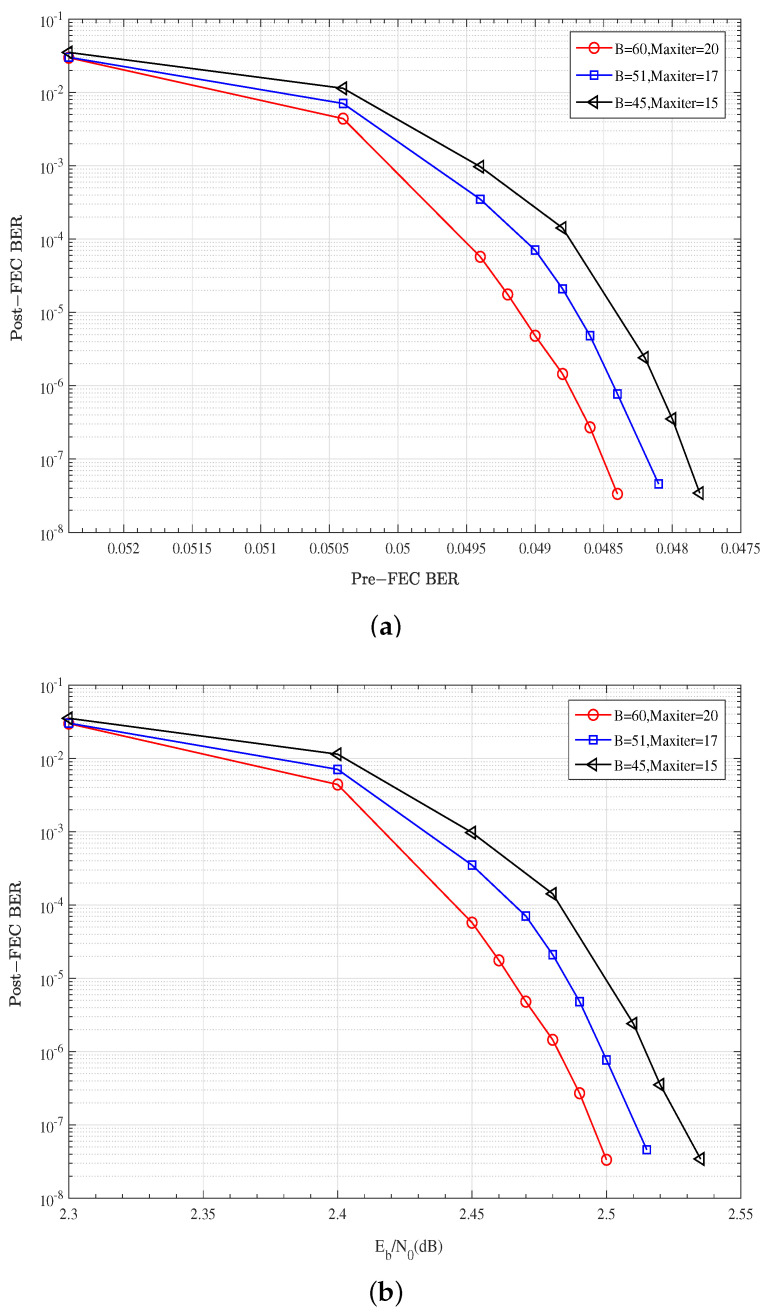
Effect of window size on the decoding performance: W=4, P=1/6, n=60, k=53, J=4, R=0.775, $\alpha_{GC}=0.75$, α=0.75: (**a**) Post-FEC BER versus Pre-FEC BER; (**b**) Post-FEC BER versus SNR.

In this subsection, the effect of window size *B* on the decoding performance is analyzed. The decoding window size is set as 60, 51 and 45, corresponding to the maximum iteration numbers of 20, 17, and 15, respectively. As shown in Figure 6, the larger the window size *B* (or maximum iteration number Maxiter, $B=Maxiter\cdot d_{v}$), the better the error correction performance. However, as shown in Equations (7) and (8), larger *B* or Maxiter also lead to a higher computation complexity and decoding latency. An appropriate decoding window size can balance the performance of these two aspects.

### 5.3. Analysis of Intra-Layer GC Node Distribution on the Error Correction Performance

**Figure 7:**
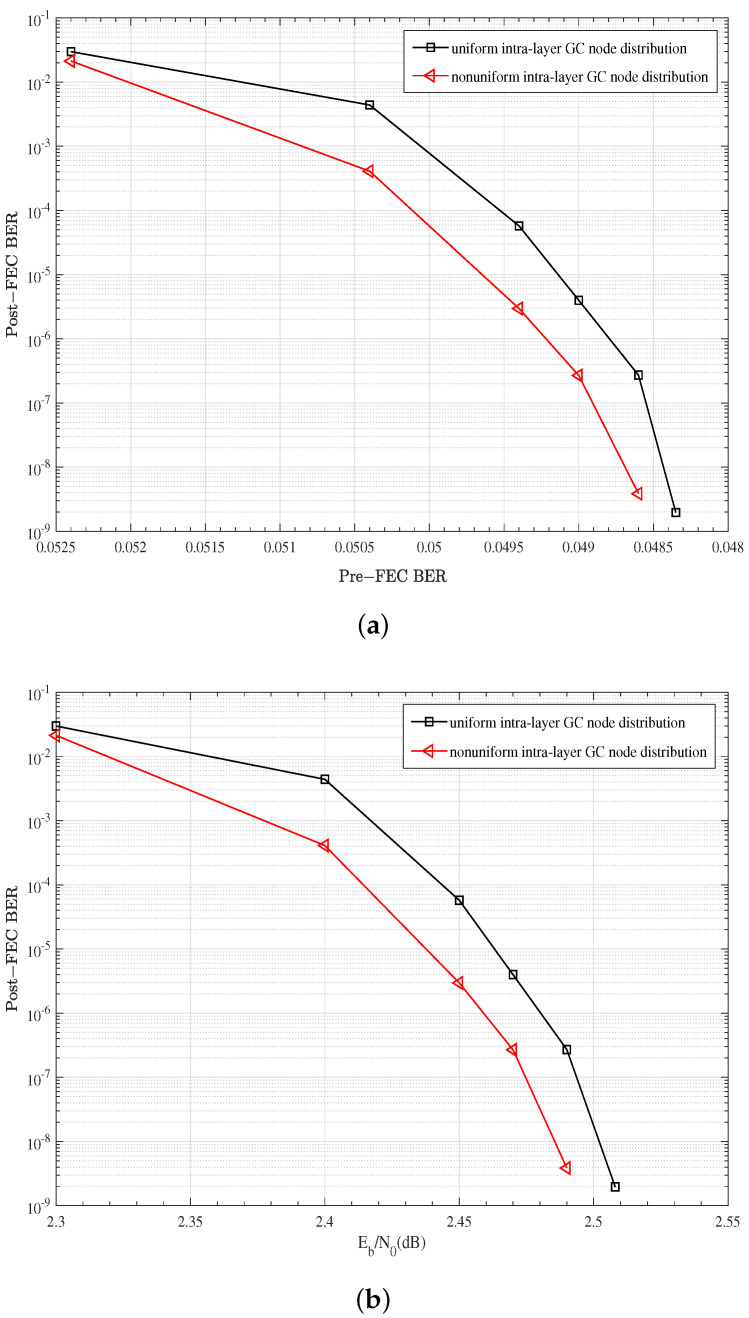
Performance comparison of different intra-layer GC node distribution: W=4, P=1/6, n=60, k=53, J=4, R=0.775, $\alpha_{GC}=0.75$, α=0.75, B=60, Maxiter=20: (**a**) Post-FEC BER versus Pre-FEC BER; (**b**) Post-FEC BER versus SNR.

In this section, the effect of intra-layer GC node distribution on the error correction performance is evaluated. We set the coding parameters as W=4, P=1/6, n=60, k=53, J=4, and R=0.775. As analyzed in Section 3.2, for the GC node code type of BCH (60,53) with W=4 and P=1/6, the uniform intra-layer GC node distribution is $N_{GC}=\left[165,\;0,\;110,\;0,\;110,\;0\right]$, and the nonuniform intra-layer distribution is $N_{GC}=\left[522,\;151,\;1215,\;95,\;288,\;0\right]$, which has more distribution types with three GC nodes and is approximately six times the uniform distribution in total. Figure 7 shows the BER performance results of the two kinds of intra-layer GC node distribution. It can be seen in Figure 7 that, with more bit nodes with two more GC check nodes, the nonuniform intra-layer GC node distribution can lead to better BER performance during the whole SNR region, and has an earlier waterfall area.

### 5.4. Analysis of Decoding Convergence Characteristics of GC Nodes and SPC Nodes

**Figure 8:**
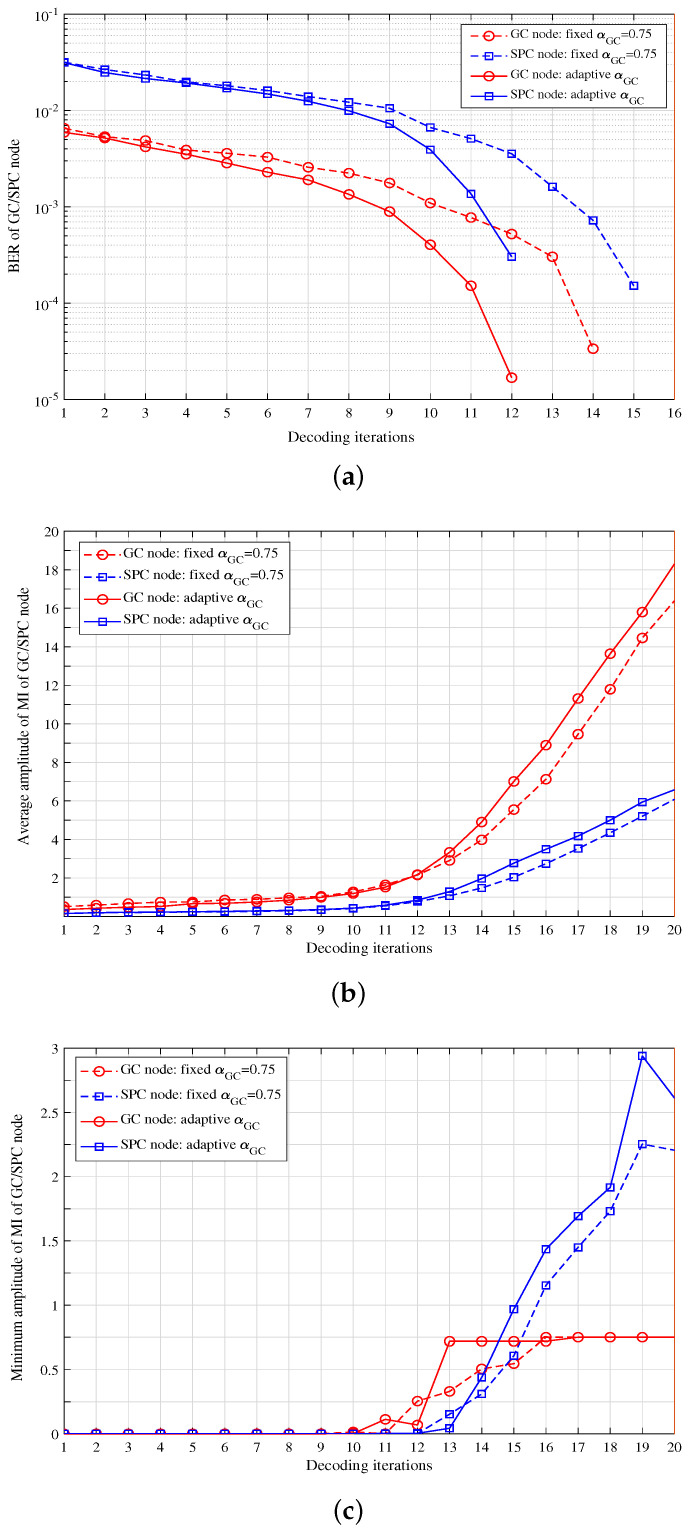
Analysis of decoding convergence characteristics of GC/SPC nodes at SNR = 2.45 dB: (**a**) Average BER performance; (**b**) Average mutual information; (**c**) Minimum mutual information.

In this section, we discuss the decoding convergence characteristics of GC nodes and SPC nodes as the decoding iterations at a certain signal-to-noise ratio (SNR). As shown in Figure 8, the convergence speed of GC node decoding is significantly faster than that of the SPC node. The error bit rate is 0.18 times (about one order of magnitude) of the SPC. The average value of the MI amplitude is approximately 2.9 times that of SPC. The minimum value of the MI amplitude is larger than that of SPC in the middle stage of decoding and gradually converges after a certain number of iterations. At the last stage of decoding, the minimum MI of GC nodes stops increasing, and becomes smaller than that of the SPC node.

On the other hand, we compare the differences in decoding convergence between the fixed $\alpha_{GC}$ and adaptive $\alpha_{GC}$ schemes, where the fixed $\alpha_{GC}$ is 0.75, and the adaptive $\alpha_{GC}$ is shown in (7). It can be found that the adaptive weighting factor scheme has a faster decoding convergence speed and higher amplitudes of mutual information.

### 5.5. Performance of Optimized HLNMS Decoding Schemes

**Figure 9:**
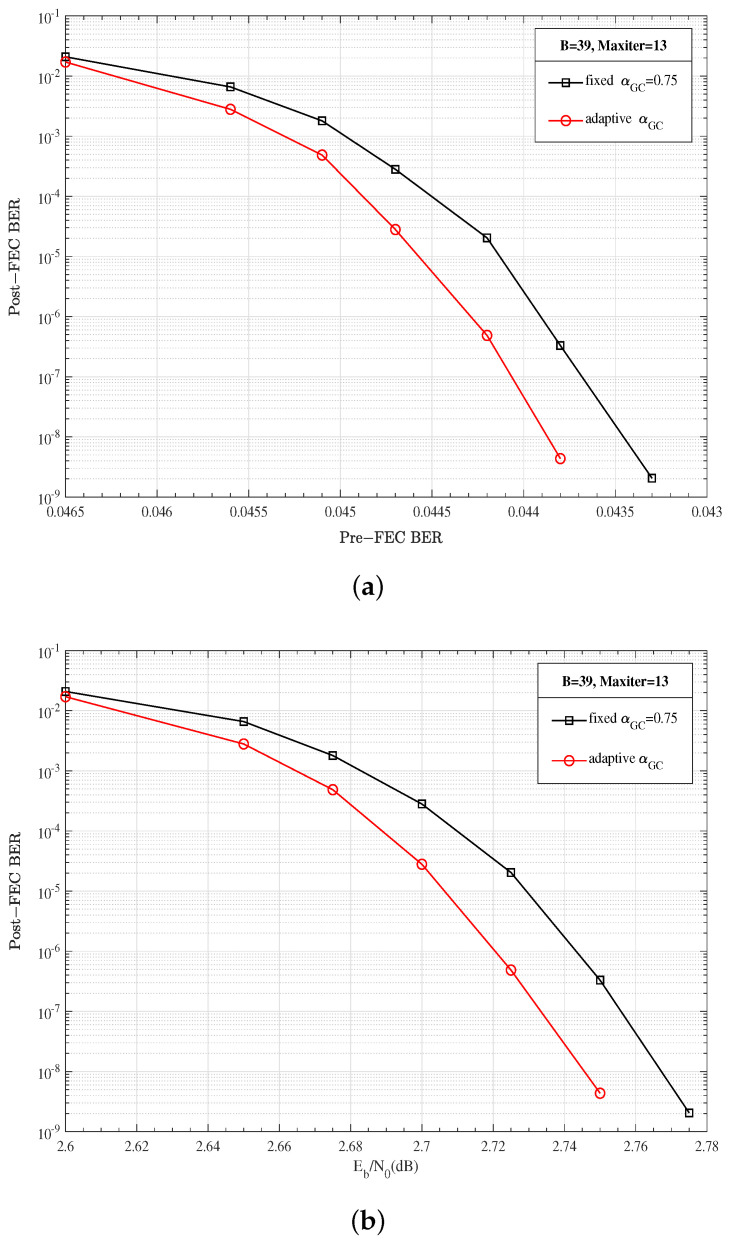
Performance comparison of the fixed weighting factor of 0.75 and the adaptive weighting factor (Scheme I): (**a**) Post-FEC BER versus Pre-FEC BER; (**b**) Post-FEC BER versus SNR.

Scheme I: Figure 8 shows the analysis of the decoding convergence characteristics of GC and SPC nodes at SNR=2.45 dB. As shown in Figure 8a, the adaptive weighting factor scheme has a faster convergence rate than the fixed weighting factor scheme. For the adaptive weighting factor scheme, i.e., Scheme I, both the GC nodes and SPC nodes have converged before the 13-th iteration. Accordingly, in Figure 9, the decoding window size is set as B=39, i.e., the maximum iteration number is set as Maxiter=13. It can be clearly observed that the adaptive $\alpha_{GC}$ scheme can achieve a lower Post-FEC BER under the same Pre-FEC BER.

**Figure 10:**
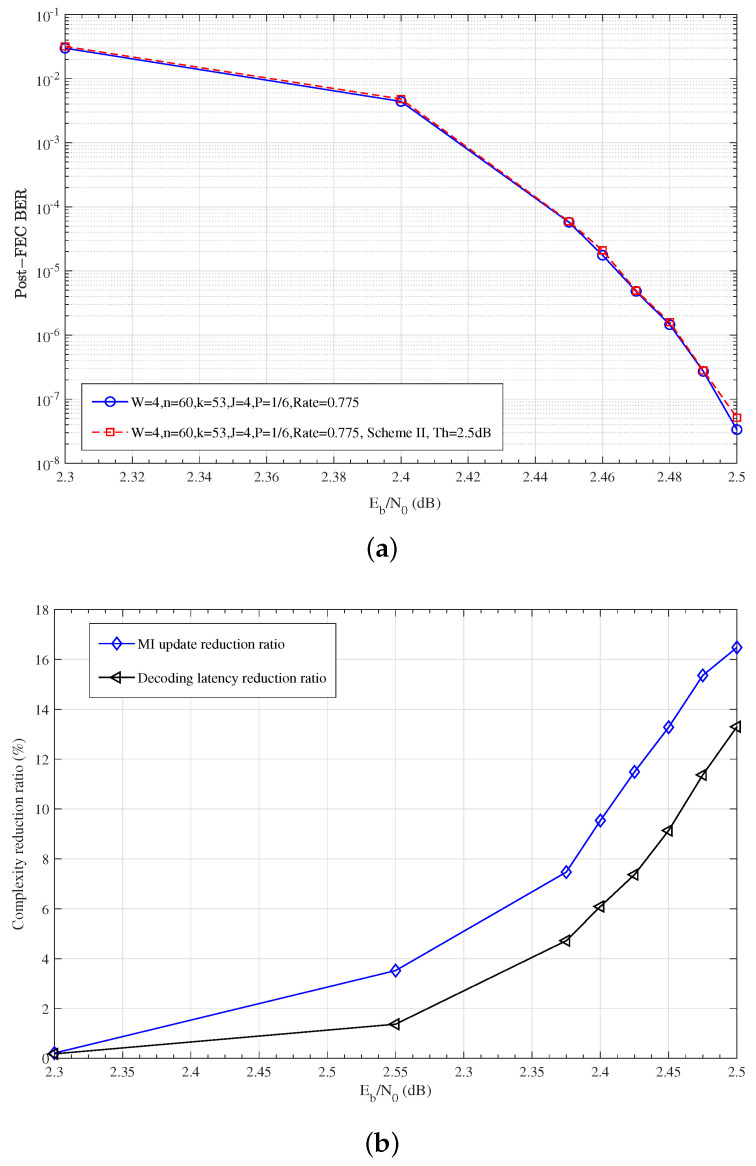
Performance of Scheme II: (**a**) Bit error rate after error correction; (**b**) Complexity reduction ratio of GC nodes; B=60, Maxiter=20, α=0.75, $\alpha_{GC}=0.75$.

Scheme II: As described in the early stopping strategy for GC node decoding in Scheme II, the selection of threshold Th is important to reduce the decoding complexity while maintaining comparable BER performance. Based on the searching method in Section 5.4, an appropriate threshold Th can be obtained. As shown in Figure 8c, the minimum amplitude of MI of the GC nodes converges at the 16th iteration for the fixed $\alpha_{GC}$ scheme. Accordingly, the initial value of Th can be set as Th_0_ = 0.75. When the tolerance parameter $BER_{Gap}$ is set as $1.0\times 10^{-5}$, the threshold Th can be searched as 2.5. It is shown in Figure 10a,b that, with the threshold of Th = 2.5, the error performance of Scheme II has not deteriorated and is comparable to that without an early stopping strategy. Moreover, both the MI update reduction ratio and the decoding latency reduction ratio for GC nodes are shown in Figure 10b, both of which increase rapidly as SNRs, and exceed 13% at the high SNR region.

### 5.6. Performance Comparisons with Prior-Arts

**Figure 11:**
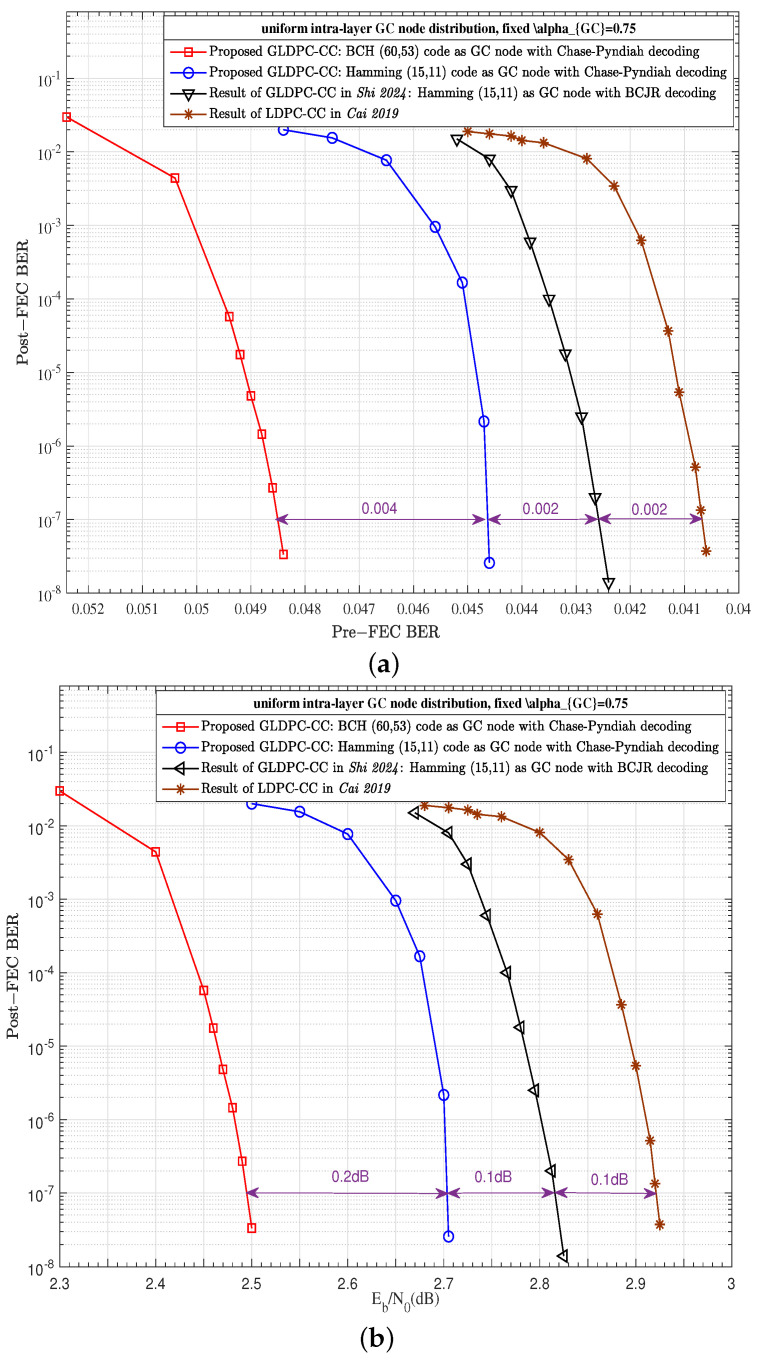
Performance comparison with the prior art: (**a**) Post-FEC BER versus Pre-FEC BER; (**b**) Post-FEC BER versus SNR [16,19].

Figure 11 shows performance comparisons with the prior art under the same coding parameters, wherein the coding rate is 0.775, the code length is 19,800 bit, and the maximum iteration is 20. Moreover, the compared schemes adopt the same uniform intra-layer GC node distribution, and the same fixed $\alpha_{GC}$ of 0.75. In Figure 11, two kinds of GC nodes of the proposed scheme are adopted, i.e., Hamming (15,11) code with GC node proportion of P=1/24 and BCH (60,53) code with GC node proportion of P=1/6, wherein the GC node of Hamming (15,11) code is the same as the GC node type of [16]. Therefore, the performance gains of about 0.1 dB come from the proposed HLNMS decoding algorithm, where the Chase–Pyndiah decoding is adopted for GC nodes. Moreover, considering that the GC node of BCH (60, 53) code has a longer code length and higher GC node proportion of P=1/6, about 0.2 dB error correction performance gain is thereby further obtained. Compared with LDPC–CC in [19], the proposed GLDPC–CC schemes demonstrates a more significant performance improvement compared to that in [16].

## 6. Conclusions

In this paper, we have proposed some optimized encoding schemes for the generalized LDPC code named as GLDPC–CCs. On the encoding side, we have provided a joint optimization method on the GC node proportion and the GC component code type, together with the optimization of intra-layer GC node distribution. Both of the optimized methods can lead to better error correction performance results. On the decoding side, we have provided two optimized decoding schemes for better performance and lower power consumption, respectively. The simulation results have verified the superiority of the proposed schemes for GLDPC–CCs over the prior art. However, the research work of this paper mainly stems from specific engineering applications. The main contributions of this work lie in the innovation of code construction and decoding algorithms, while the contributions to the theoretical analysis are relatively insufficient. Accordingly, in the next stage of our research, we plan to conduct an in-depth theoretical analysis of the existing research based on some analytical methods, including the analysis of the minimum distance, the decoding threshold, and the error floor of the proposed codes, as well as the complexity analysis of the proposed decoding algorithms, etc.

## Figures and Tables

**Table 1:** Component code type and parameter comparison.

W	n	k	P	BCH Code	Truncated Number J	Original BCH Code	Rate
1	15	11	1/24	(15,11)	0	(15,11)	0.775
2	30	25	1/12	(30,25)	1	(31,26)	0.775
2	30	20	1/32	(30,20)	1	(31,21)	0.775
4	60	54	1/4	(60,54)	3	(63,57)	0.775
4	60	53	1/6	(60,53)	4	(63,57)	0.775
4	60	42	1/28	(60,42)	3	(63,45)	0.775
8	120	106	1/6	(120,106)	7	(127,113)	0.775
8	255	231	17/56	(255,231)	0	(255,231)	0.775

**Table 2:** The inter-layer distribution types of GC nodes.

Type	Layer of GC Nodes	Number of GC Nodes	Number of SPC Nodes
1	Layer 1 + Layer 2	2	1
2	Layer 1 + Layer 2 + Layer 3	3	0
3	Layer 2 + Layer 3	2	1
4	Layer 2 + Layer 3 + Layer 1	3	0
5	Layer 3 + Layer 1	2	1
6	Layer 3 + Layer 1 + Layer 2	3	0

## Data Availability

No new data were created or analyzed in this study. Data sharing is not applicable to this article.
